# Different Impact of Pretransplant Anti-HLA Antibodies Detected by Luminex in Highly Sensitized Renal Transplanted Patients

**DOI:** 10.1155/2013/738404

**Published:** 2013-09-05

**Authors:** Isabel Pérez-Flores, Jose Luis Santiago, Natividad Calvo-Romero, Alberto Barrientos-Guzmán, Ana Isabel Sánchez-Fructuoso

**Affiliations:** Immunology Department, Hospital Clínico San Carlos, Instituto de Investigación Sanitaria del Hospital Clínico San Carlos (IdISSC), Martín Lagos s/n, 28040 Madrid, Spain

## Abstract

It is well know that anti-HLA antibodies are an important obstacle in kidney transplantation. Our aim was to study the clinical impact of pretransplant donor specific anti-HLA antibodies (HLA-DSA), in highly sensitized (HS) patients. We analyzed retrospectively the day-of-transplant sera by Luminex Single Antigen Assay (LSA) in HS patients, and the results were correlated with episodes of humoral and cellular rejection as well as with graft and patient survival. All HS subjects received the same induction therapy and rejection episodes were biopsy proven. Thirteen patients (56.5%) preformed HLA-DSA, and we observed higher incidence of acute rejection in aforementioned patients than in the pre-transplant negatives DSA recipients (77% versus 30%, *P* = 0.03). The one-year graft survival was significantly reduced in positive pre-transplant HLA-DSA patients (60% versus 100%, *P* = 0.01 Breslow). The positive predicted value of HLA-DSA in relation to rejection reached 100% if patients lost their previous graft in the first year after transplant. Among anti-HLA antibodies present in patients before transplant, HLA-DSA were significantly associated with high risk of acute humoral and cellular rejection and reduced graft survival in posttransplant outcome. The negative impact of these antibodies was even higher when patients suffered an early loss of the previous transplant.

## 1. Introduction

A best knowledge of immune system and the introduction of several immunosuppressive agents have led to reduce the incidence of acute rejection (AR) but had a limited impact on long-term allograft survival [1]. It is well known that the waiting time for highly sensitized (HS) patients is much longer than for who are not sensitized and a longer time on dialysis has demonstrated to be a negative impact on graft survival [2]. Patients with high levels of panel reactive antibodies (PRA) have difficulty achieving a negative crossmatch and when these subjects are transplanted, they have a high risk of developing an acute rejection, mainly acute humoral or antibody-mediated rejection (AMR), and the graft survival is inferior to nonsensitized recipients [3].

Since the number of retransplanted patients is increased year by year, the study of anti-HLA antibodies using a method which can improve sensitivity and specificity to detect donor specific anti-HLA-antibodies (DSA) would be useful in order to avoid the acute rejection. Luminex technology is one of the solid assays which have been developed in the last few years. This technology is able to detect lower levels of alloantibody and to define them more accurately than the conventional complement-dependent cytotoxicity (CDC) assay [4–6]. Despite the negative impact of *de novo *DSA has been well established, the determination of the clinical relevance of the preformed anti-HLA antibodies is today under consideration [7, 8]. Published studies to date have been controversial [8–12]. Several authors have pointed out that the successful allocations of suitable renal allograft have been possible in sensitized patients as a result of the detection and definition of alloantibodies with a higher degree of sensitivity and specificity [13, 14]. However, another research even considered that single antigen assays could be a new technological barrier to transplantation in immunized recipients [15].

Our aim was to determine among all of the anti-HLA antibodies, the effect of pre-transplant (pre-Tx) DSA in clinical outcome. For that purpose we selected HS patients and we analyzed retrospectively the day of transplant sera by Single Antigen in order to elucidate how predictive were the DSA present in the receptors before kidney transplantation in acute rejection and allograft survival. 

## 2. Subjects and Methods

### 2.1. Study Design

This is a retrospective study to analyze the role of preformed antibodies in solid organ rejection. In order to guarantee the presence of DSA in pre-transplant sera, we selected all HS patients (defined by historical PRA >75%) transplanted in a short period of time (26 months). 

The induction protocol used in all HS patients was the same: thymoglobulin (1–1.2 mg/kg pre-transplant and 4–7 doses after-transplant 0.25–0.75 mg/kg/daily), tacrolimus (FK, 0.05 mg/kg pre-transplant and every 12 hours after transplant), mycophenolate mofetil (MMF, 1000 mg pretransplant, and 500 mg/12 hours after transplant) and steroids: methylprednisolone (MPN 250 mg pretransplant and 1 mg/kg/daily after transplant, with progressive doses being decreased to obtain 20 mg first week and 10 mg in the 2nd and 3rd months). No desensitization protocol was performed.

All patients received steroids pulses as a first-line treatment for cellular rejection, and in the most severe cases we added thymoglobulin. In patients with AMR, the treatment was based on intravenous immunoglobulin (IVIG, 2 g/kg of weight), and when there was severe rejection (Banff grade II or III), we added plasmapheresis or rituximab (1–4 doses of 375 mg/m2 in 7/10 cases). 

### 2.2. HLA Antibody Analysis

The analysis of anti HLA class I antibodies was carried out by Single Antigen (Tepnel Lifecodes LSA Stamford CT, Diagnostica Longwood) according to manufacturer's instruction. Luminex 100 IS 2.3 system was used for data acquisition and analysis and all beads with mean fluorescence intensity (MFI) above 3000 were considered as positives. The determination of anti HLA class II antibodies was carried out by Lifecodes Class II ID, which is a Luminex assay for detection of IgG panel reactive antibodies to HLA class II molecules.

### 2.3. Statistical Analysis

Normally distributed variables are expressed as mean ± standard deviation (SD) and nonnormally distributed variables are expressed as median and interquartile range (IQR). Association between qualitative variables was evaluated by chi-square test or Fisher's exact test when necessary. Effect was calculated as risk ratio (RR) and 95% confidence interval. Quantitative variables by groups were evaluated with *t*-Student or *U* Mann-Whitney. Level of significance was *P* < 0.05 SPSS v. 13.0 software was used in statistical analysis (Chicago, Illinois).

## 3. Results

**Figure 1:**
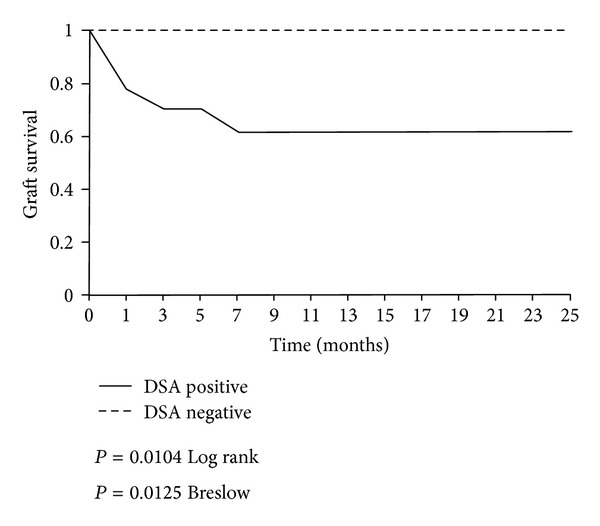
Allograft survival depending on positivity of DSA (median followup: 14 months).

We selected 23 HS patients out of 191 subjects transplanted in our Renal Transplant Unit between 2007 and 2008 and followed during 25 months (median of followup 14 months, see Figure 1). Donors' characteristics, HLA mismatch, and cold ischemia time were similar between high and low immunological risk patients and when we analyzed the HS patients separately by presence or absence of preformed DSA (Table 1). From 23 HS recipients, 22 were retransplanted and the 56% showed HLA-DSA in the the day-of-transplant sera with mean fluorescence intensity (MFI) of 6000 (3345-8990). 

The incidence of AR and the death censored allograft one year survival was significantly different between non-HS group and the HS patients (Table 1). Interestingly, we find differences in AR and death censored allograft one year survival when we compare HS patients with and without preformed DSA (Table 1). In fact, these differences are due to HS patients with preformed DSA because only these recipients were significantly different from non-HS patients (*P* = 0.002 for AR and *P* < 0.001 for death censored 1 year allograft survival) and no differences were found between HS patients with nonpreformed DSA and non HS-patients (*P* = 0.6 and *P* = 0.7, resp.). 

In Table 2, we analyzed the main known risk factors involved in acute rejection in the HS patients group. Only one out of nine factors studied was statistically different when we compared patients who suffered humoral and/or cellular acute rejection with recipients free from rejection. This factor, the presence of pre-transplant donor anti-HLA antibodies, was even more critical in risk of graft failure than the PRA class I or class II values (Table 2). 

We also performed a Kaplan-Meier survival analysis, and the presence of DSA in the pre-transplant sera correlates with a pour graft outcome (60% versus 100%; Figure 1) In fact, six patients were transplantectomyzed (mean 25 days after transplant) and all of them were DSA positives in the pre-transplant sera. 

**Figure 2:**
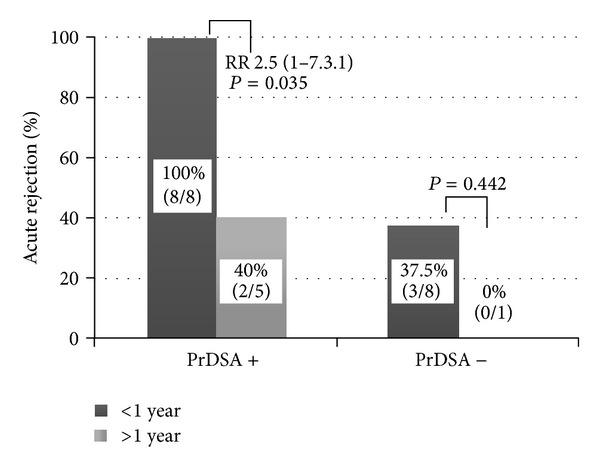
Relationship between early loss of previous transplant, preformed DSA against the new graft, and incidence of rejection. PrDSA: performed DSA. Duration of previous renal transplant: < or >1 year.

Although 77% of patients who presented acute rejection episodes showed DSA pre-transplant, there were 3 out 13 patients with pre-transplant DSA without any type of acute rejection (positive predictive value = 70%; *P* = 0.03). In order to improve this predictive value, we analyzed the characteristics of all data which could be implied in acute rejection. Neither induction treatment (it was the same one for all the patients) nor immunization events, 22 out of 23 had a previous transplant, could be considered as critical predictive factor. We also analyzed the number, class and MFI value of the pre-transplant DSA and no differences were found between patients with and without acute rejection. Regarding clinical parameters, we considered the number of previous transplant, elapsed time after the last transplant, whether patient had suffered AR in the previous transplant and the survival of the last graft. Interestingly, only the early loss of previous graft showed a significant statistically association with the presence of preformed DSA and improved its predictive value of rejection. In fact, when patients lost the previous transplant before first year and preformed DSA against the new graft, the presence of AR was 100% (Figure 2).

## 4. Disscusion

Whereas CDC crossmatch positivity is an absolute contraindication to kidney transplantation, the clinical relevance of DSA detected by Luminex technology in patients with CDC crossmatch negative remains today unclear. It is well knows that Luminex solid assay has a higher sensitivity than the CDC, hence the number of anti-HLA antibodies positive patients increases when this technique is used and therefore the group of HS patients is also higher [15]. 

The major limitation of our study is the size of our cohort that can compromise the statistical power; however, due to the fact that all subjects were HS patients transplanted in a short period of time with identical immunosuppression protocol allow us to consider that conclusions of our study could be interesting. Moreover, the majority of the cases were biopsied during the followup. 

Patients with positives DSA in the pre-transplant sera showed a worse prognosis than subjects without DSA. Additional to elevated incidence of acute rejection, this group of patients had a higher graft loss than the aforementioned control group. Opposite to our finding, Gupta et al. [10] have reported that acute rejection episodes, delayed graft function, and one-year graft survival was similar between patients with and without HLA specificities defined by solid phase assay. It could be explained by the fact that in their group of patients with HLA antibodies before transplant, only 4 out 16 patients with DSA and 4 out of 22 patients without DSA (NDSA) had a historic HLA class I PRA >50% (cut-off value that they considered patients as HS). However, all our patients had a historic PRA >75% that mean all of them are HS subjects and therefore the pre-transplant immunologic background was similar in all patients under study. This presence of high levels of anti-HLA antibodies guarantees the presence of pre-transplant DSA even to a subtyping allele likelier than in patients with lower PRA values in which antibodies against to a specific HLA-allele could not cover all subtypes of this allele included the next donor's alleles. 

In a large retrospective study, Süsal et al. [11] have reported also no association of kidney graft loss with anti-HLA antibodies detected by LSA. The main limitation of this study was exposed by authors because they have no biopsy data for confirmation of immunological graft loss, hence this group includes all causes of graft failure not only the immune system mediated. It is clear that the implication of pre-transplant DSA in kidney allograft loss must be focused in causes due to immune system. 

Supporting our results that pre-transplant DSA is a risk factor of rejection, Loupy et al. and Amico et al. have reported that these preformed antibodies correlate with a high incidence of subclinical AMR [9, 16] and recently, Kanter Berga et al. [17] find that pre-Tx DSA were associated with acute vascular and chronic rejection and poorer graft outcome.

In conclusion, although the positive predictive value of DSA by LSA is 70% in our study considering the overall cohort, when we add the qualitative and indirect data of early loss of previous transplant, the potential of worse graft outcome increases to 100% (all patients with humoral type III rejection and graft loss belong to this group). In these cases we would be able to talk about “DSA susceptible patients” who are more sensitive to foreign HLA antigens and usually the graft loss occurs in a period of time lower than a year. We considered that the presence of both pre-transplant DSA and early loss of the previous organ is a high risk factor for rejection. In case that DSA not disappear in a reasonable period, desensitization protocols (e.g., plasma exchange and intravenous immunoglobulin) could be necessary to transplant these patients and to avoid the almost sure nephrectomy.

## Figures and Tables

**Table 1:** Demographic and clinical characteristics.

	Non-HS	HS	*P*	HS Pre-Tx DSA	HS non-DSA	*P*
	*n* = 167	*n* = 23		*n* = 13	*n* = 10	
Donor						
Gender (% male)	69%	68%	0.9	71%	68%	0.7
Age (years, mean ± SD)	40 ± 15	39 ± 10	0.8	41 ± 12	40 ± 8	0.8
Recipient						
Gender (% male)	64%	62%	0.8	61.5%	30%	0.1
Age (years, mean ± SD)	50 ± 13	44 ± 10	0.03	43 ± 12	44 ± 16	0.9
HLA mismatch (median, IQR)	4 (3–5)	4 (3–5)	—	4 (3–5)	4 (3–5)	—
Cold ischemia time (hours, mean ± SD)	19 ± 5	18 ± 6	0.4	19 ± 5	17 ± 7	0.4
Delayed graft function (DGF)*	62%	65%	0.7	67%	63%	0.5
Acute rejection (AR)	33%	57%	0.03	77%	30%	0.03
Death censored 1 year allograft survival	96%	78%	0.008	46%	100%	0.007

*DGF was defined as the need for dialysis during the first week after transplant.

**Table 2:** Univariable study of risk factors of acute rejection in HS group.

	Acute rejection	Non rejection	*P*
	(*n* = 13)	(*n* = 10)	
Age of donor (years, mean ± SD)	40 ± 9	42 ± 5	0.5
Age of recipient (years, mean ± SD)	44 ± 10	43 ± 13	0.8
Number of transplant (0/1/2/3)	0/9/3/1	1/8/1/0	0.4
HLA mismatch (median, IQR)	4 (4-5)	4 (4-5)	0.7
DCD* (%)	54%	50%	0.6
Cold ischemia time (hours, mean ± SD)	18 ± 6	19 ± 7	0.7
Maximum PRA	83 ± 19	65 ± 25	0.07
Current PRA	46 ± 35	25 ± 24	0.2
PRA class I	69 ± 24	81 ± 14	0.3
PRA class II	44 ± 33	28 ± 33	0.3
Pre-transplant DSA (%)	10 (77%)	3 (30%)	0.03
MFI** of DSA (mean ± SD)	6188 ± 1670	5824 ± 2533	0.8

*DCD: donation after cardiac death.

**MFI: mean fluorescence intensity.

## References

[1] Meier-Kriesche HU, Ojo AO, Hanson JA (2000). Increased impact of acute rejection on chronic allograft failure in recent era. *Transplantation*.

[2] Meier-Kriesche HU, Kaplan B (2002). Waiting time on dialysis as the strongest modifiable risk factor for renal transplant outcomes: a paired donor kidney analysis. *Transplantation*.

[3] Piazza A, Pocci E, Borrelli L (2001). Impact of donor-specific antibodies on chronic rejection occurrence and graft loss in renal transplantation: posttransplant analysis using flow cytometric techniques1. *Transplantation*.

[4] Bray RA, Gebel HM (2009). Strategies for human leukocyte antigen antibody detection. *Current Opinion in Organ Transplantation*.

[5] El-Awar N, Lee J, Terasaki PI (2005). HLA antibody identification with single antigen beads compared to conventional methods. *Human Immunology*.

[6] Pei R, Lee JH, Shih NJ, Chen M, Terasaki PI (2003). Single human leukocyte antigen flow cytometry beads for accurate identification of human leukocyte antigen antibody specificities. *Transplantation*.

[7] Gebel HM, Moussa O, Eckels DD, Bray RA (2009). Donor-reactive HLA antibodies in renal allograft recipients: considerations, complications, and conundrums. *Human Immunology*.

[8] Lefaucheur C, Suberbielle-Boissel C, Hill GS (2009). Clinical relevance of preformed HLA donor-specific antibodies in kidney transplantation. *Contributions to Nephrology*.

[9] Amico P, Hönger G, Mayr M, Steiger J, Hopfer H, Schaub S (2009). Clinical relevance of pretransplant donor-specific HLA antibodies detected by single-antigen flow-beads. *Transplantation*.

[10] Gupta A, Iveson V, Varagunam M, Bodger S, Sinnott P, Thuraisingham RC (2008). Pretransplant donor-specific antibodies in cytotoxic negative crossmatch kidney transplants: are they relevant?. *Transplantation*.

[11] Süsal C, Ovens J, Mahmoud K (2011). No association of kidney graft loss with human leukocyte antigen antibodies detected exclusively by sensitive luminex single-antigen testing: a collaborative transplant study report. *Transplantation*.

[12] Van Den Berg-Loonen EM, Billen EVA, Voorter CEM (2008). Clinical relevance of pretransplant donor-directed antibodies detected by single antigen beads in highly sensitized renal transplant patients. *Transplantation*.

[13] Bingaman AW, Murphey CL, Palma-Vargas J, Wright F (2008). A virtual crossmatch protocol significantly increases access of highly sensitized patients to deceased donor kidney transplantation. *Transplantation*.

[14] Vaidya S, Partlow D, Susskind B, Noor M, Barnes T, Gugliuzza K (2006). Prediction of crossmatch outcome of highly sensitized patients by single and/or multiple antigen bead luminex assay. *Transplantation*.

[15] Gupta A, Sinnott P (2009). Clinical relevance of pretransplant human leukocyte antigen donor-specific antibodies in renal patients waiting for a transplant: a risk factor. *Human Immunology*.

[16] Loupy A, Suberbielle-Boissel C, Hill GS (2009). Outcome of subclinical antibody-mediated rejection in kidney transplant recipients with preformed donor-specific antibodies. *American Journal of Transplantation*.

[17] Kanter Berga J, Sancho Calabuig A, Gavela Martinez E (2012). Pretransplant donor-specific HLA antibodies detected by single antigen bead flow cytometry: risk factors and outcomes after kidney transplantation. *Transplantation Proceedings*.

